# Varenicline and counseling for vaping cessation: a double-blind, randomized, parallel-group, placebo-controlled trial

**DOI:** 10.1186/s12916-023-02919-2

**Published:** 2023-07-05

**Authors:** Pasquale Caponnetto, Davide Campagna, Jasjit S. Ahluwalia, Christopher Russell, Marilena Maglia, Paolo Marco Riela, Carmelo Fabio Longo, Barbara Busa, Riccardo Polosa

**Affiliations:** 1 Centre for the Prevention and Treatment of Tobacco Addiction (CPCT), University Teaching Hospital “Policlinico-Vittorio Emanuele”, University of Catania, Catania, Italy; 2 Center of Excellence for the Acceleration of HArm Reduction (CoEHAR), University of Catania, Catania, Italy; 3 Department of Science of Education, Section of Psychology, University of Catania, Catania, Italy; 4 UOC MCAU, University Teaching Hospital “Policlinico-Vittorio Emanuele”, University of Catania, Catania, Italy; 5 Department of Clinical & Experimental Medicine, University of Catania, Catania, Italy; 6 Brown University School of Public Health and Alpert School of Medicine, RI Providence, USA; 7 Russell Burnett Research & Consultancy Ltd, Glasgow, UK; 8 ECLAT Srl, Spin-off of the University of Catania, Catania, Italy; 9 Department of Mathematics and Informatics, University of Catania, Catania, Italy; 10 UOC Farmacia Ospedaliera, ARNAS Hospital “Garibaldi”, Catania, Italy

**Keywords:** Vaping cessation, Vaping cessation counseling, E-cigarettes, Varenicline, Randomized controlled trial

## Abstract

**Background:**

Vaping cessation is virtually unexplored. The efficacy and safety of varenicline for vaping cessation has not been studied and rigorous research is required to advance best practice and outcomes for people who use electronic cigarettes (EC) and want to quit. The objective is to evaluate the efficacy and safety of varenicline (1 mg BID, administered for 12 weeks, with follow-up to week 24) combined with vaping cessation counseling in exclusive daily EC users intending to quit vaping.

**Methods:**

Design: Double-blind, randomized, parallel-group, placebo-controlled trial.

Setting: The study took place at a University-run smoking cessation center.

Participants: People who exclusively use ECs daily and intend to quit vaping.

Intervention: A total of 140 subjects were randomized to either varenicline (1 mg, administered twice daily for 12 weeks) plus counseling or placebo treatment (administered twice daily, for 12 weeks) plus counseling. The trial consisted of a 12-week treatment phase followed by a 12-week follow-up, nontreatment phase.

Main outcomes and measures: The primary efficacy endpoint of the study was biochemically validated continuous abstinence rate (CAR) at weeks 4 to 12. Secondary efficacy end points were CAR at weeks 4 to 24 and 7-day point prevalence of vaping abstinence at weeks 12 and 24.

**Results:**

CAR was significantly higher for varenicline vs placebo at each interval: weeks 4–12, 40.0% and 20.0%, respectively (OR = 2.67, 95% CI = [1.25–5.68], *P* = 0.011); weeks 4–24, 34.3% for varenicline with counseling and 17.2% for placebo with counseling (OR = 2.52, 95% CI = [1.14–5.58], *P* = 0.0224). The 7-day point prevalence of vaping abstinence was also higher for the varenicline than placebo at each time point. Serious adverse events were infrequent in both groups and not treatment-related.

**Conclusions:**

The findings of the present RCT indicate that inclusion of varenicline in a vaping cessation program for people who use electronic cigarettes and intending to quit may result in prolonged abstinence. These positive findings establish a benchmark of intervention effectiveness, may support the use of varenicline combined with counseling in vaping cessation programs, and may also help guiding future recommendations by health authorities and healthcare providers.

**Trial registration:**

The study has been registered in EUDRACT with Trial registration ID: 2016-000339-42.

**Supplementary Information:**

The online version contains supplementary material available at 10.1186/s12916-023-02919-2.

## Background

Electronic cigarettes (EC) are becoming increasingly popular with people who smoke worldwide [1–3]. Users report buying them mainly to help abstain from smoking cigarettes, to relieve cigarette withdrawal symptoms, to save money, and to continue to have a “smoking” experience but with reduced health risks [4, 5].

Because ECs do not contain tobacco and do not rely on combustion to operate, the aerosol generated by ECs contains fewer and substantially lower levels of harmful and potentially harmful chemicals compared to combustible tobacco cigarettes under normal conditions of use [6–8]. For this reason, ECs have been proposed as a tool for reducing harm from cigarette smoking [9–11]. Evidence from randomized controlled trials, observational studies, and population data converge on showing that EC use (“vaping”) is an effective method of smoking cessation, with daily vaping being more effective than less frequent use [12–15]. Nonetheless, the long-term health effects of combustion-free nicotine products are still not fully known and require investigation. The potential health impact of ECs has been addressed in two recent review articles, with conflicting conclusions [16, 17]. Moreover, perceptions of ECs being equally or more harmful than combustible cigarettes have increased in the past few years, raising concern among people who use ECs about the potential health risks of vaping and long-lasting nicotine addiction [18, 19].

In addition to increasing concerns about the potential health risks of vaping, growing interest in quitting vaping has also been linked to the experience of some adverse physical effects (e.g., dry mouth, cough), the rising cost of vaping, and the need to break dependence on vaping products [20–22].

Although guidelines on best management for the cessation of combustible cigarettes are available [23, 24], there are no evidence-based recommendations to assist EC users intending to quit vaping, and it is unclear whether smoking cessation guidelines can be extrapolated to vaping products. In particular, there are no studies of the efficacy of medications approved for smoking cessation by the US Food and Drug Administration (FDA) for aiding vaping cessation. The efficacy and safety of varenicline for vaping cessation has not been studied and rigorous research is required to guide the decisions of health authorities and healthcare providers.

The aim of this double-blind randomized placebo-controlled trial was to evaluate the efficacy and safety of varenicline (1 mg BID, administered for 12 weeks, and followed to week 24) combined with vaping cessation counseling in exclusive daily EC users intending to quit vaping.

## Methods

### Participants

Exclusive EC users who were vaping daily and intending to quit vaping were screened for inclusion in this study.

The specific eligibility criteria were:

- Inclusion criteria: (a) ≥ 18 years of age; (b) exclusive daily EC use for ≥ 12 months; (c) at least one serious quit vaping attempt (defined as complete abstinence for at least 24 h) in the past; (d) willingness to quit vaping, confirmed by a “YES” response to each of two questions “Do you plan to quit vaping within the next 30 days?” and “Do you wish to participate in a vaping cessation program?”; (e) self-reported reduction in vape consumption by at least 50% before committing to target quit date (TQD) (this instruction is given at screening).
- Exclusion criteria: (a) current diagnosis of mental illnesses including major depression, psychosis, or bipolar disorder that were diagnosed and treated by psychiatrists or clinical psychologists; (b) history of alcoholism or drug/chemical abuse within 12 months prior to screening; (c) known medical condition that, in the opinion of the investigators, would compromise subjects’ safety or participation; (d) currently pregnant or breast feeding or intending to become pregnant during the trial; (e) use of vaping products containing zero nicotine.

**Fig. 1:**
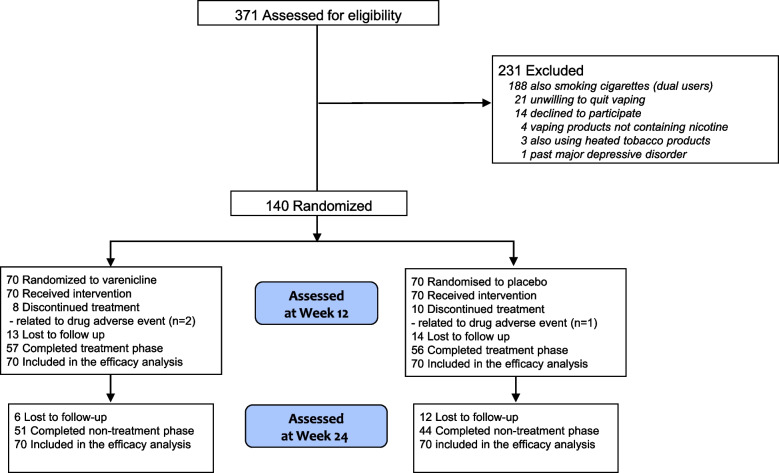
Study flow diagram of study participants

Eligible subjects were recruited from local vape shops, databases of people who previously smoked who attended a local smoking cessation center (Centro per la Prevenzione e Cura del Tabagismo (CPCT), University of Catania) and stopped smoking by switching to ECs, databases of people who previously smoked who took part in CoEHAR (CoEHAR, University of Catania) sponsored tobacco harm reduction and switching studies, social networks, WhatsApp chat of undergraduates and postgraduates of the University of Catania, and word of mouth among relatives and friends of study participants. The flow diagram of subjects is shown in Fig. 1, according to the Consolidated Standards of Reporting Trials (CONSORT) reporting guideline. Screening started in April 2018 (first subject first visit was conducted in July 2018) and concluded in February 2020. Last subject last visit was completed in September 2020).

The study was performed in accordance with ethical principles that have their origin in the Declaration of Helsinki and are consistent with ICH/GCP applicable regulatory principles. The local ethical review board of the Azienda Ospedaliero Universitaria Policlinico-Vittorio Emanuele (part of the Hospital Trust of Università di Catania) reviewed and approved the study protocol (approval reference number: n.88/2016/PO, 11/07/2016). All participants provided written informed consent prior to participating in the trial. The study has been registered in EUDRACT with Trial registration ID: 2016-000339-42. Due to the unexpected issue of poor recruitment of people who smoke and use ECs, a change in the protocol was deemed necessary. The protocol amendment was reviewed and approved to include single users intending to quit vaping and dual users intending to quit smoking (n. 91/20l8/EMPO, 15/10/2018); data from dual users will be analyzed and presented in a separate paper.

### Study design and study assessment

This was a double-blind, randomized, parallel-group, placebo-controlled trial (RCT) to investigate efficacy and safety of varenicline (1 mg, administered twice daily for 12 weeks) versus placebo (administered twice daily, for 12 weeks) together with counseling for vaping cessation in exclusive daily EC users intending to quit vaping.

**Fig. 2:**
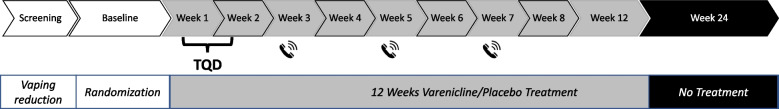
Varevape single — study design. Exclusive daily EC users who intended to quit vaping were randomized to receive either varenicline, 1 mg, twice daily for 12 weeks or matched placebo for 12 weeks. Subjects were prospectively reviewed for up to 24 weeks during which vaping habits, questionnaire answers, adverse events, and vital signs were assessed at each visit. The telephone symbol indicates telephone contact

The trial consisted of a 12-week treatment phase directly followed by a 12-week non-treatment phase (Fig. 2). The study took place at Centro per la Prevenzione e Cura del Tabagismo (CPCT), the University-run smoking cessation center.

### Randomization and trial interventions

Eligible subjects were randomly assigned to either varenicline or placebo in a 1:1 ratio by using a computer-generated, 5-block randomization scheme. Varenicline (0.5 mg tablet) and matched placebo tablets were supplied by Pfizer and randomized by the hospital pharmacy. Participants assigned to varenicline were titrated to full dose by the time of their TQD (0.5 mg/day for 2–3 days, 0.5 mg twice daily for 4–5 days; then 1 mg twice daily for 11 weeks). All subjects in both treatment groups received the same vaping cessation counseling throughout the whole duration of the study. One-on-one counseling was provided at each visit for a total of 10 min by clinical psychologists with experience in nicotine dependence and vaping behaviors. Subjects returned blister cards at each programmed visit and a dosage record was registered.

At screening, subjects who reported vaping daily and an intention to quit vaping were assessed for eligibility. Before leaving they were instructed to reduce the average daily vape use by at least 50% before making an appointment to attend the baseline visit (V1).

At the baseline visit (V1), eligibility criteria were reassessed and subjects were randomized (1:1) to either varenicline plus vaping cessation counseling or placebo plus vaping cessation counseling. The list for treatment randomization was generated using SAS software (SAS Institute). The size of the blocks was a variable of 5, and the sequence of blocks was randomized and blinded. The following data were recorded at V1: sociodemographic characteristics, medical history, smoking and vaping history (including EC type, e-liquid flavor, and nicotine concentration), and motivation (and reasons) for quitting vaping, vaping/nicotine consumption (assessed by modified Nicotine Use Inventory, mNUI), exhaled carbon monoxide (eCO) levels, blood pressure, heart rate, weight/body mass index (BMI), questionnaires’ scores (Penn State Electronic Cigarette Dependence Index (PSECDI)); Beck Depression Inventory-II (BDI-II)); Beck Anxiety Inventory (BAI)); Minnesota Nicotine Withdrawal Scale (MNWS)), level of motivation to quit vaping (assessed by visual analog score (VAS)), and adverse events. Participants received their first vaping cessation counseling session and were instructed to set a target quit date (TQD) that was within the next 10 days. Prior to check-out, subjects were given a full week’s supply of the assigned treatment (either varenicline or placebo, depending on the treatment arm). Study drugs were dispensed in accordance with the plan (Additional file 1: Table S1).

After V1, subjects were invited to return to the clinic on a weekly basis for the following 12 weeks (V2-V10), except for visits 4, 6, and 8 (telephone contact). At each visit, subjects underwent vaping cessation counseling. Modified NUI, eCO levels, blood pressure, heart rate, weight/BMI (only at Week-12 visit), MNWS (only at week-1, 2, 4, 6, 8, 12 visit), and adverse events were recorded in the CRF at each study visit. At week-4 (V5), week-6 (V7), week-8 (V9), and week-12 (V10), saliva samples were collected for cotinine assessment. Study drugs were dispensed before check-out in accordance with the plan (Additional file 1: Table S1).

The study was continued in the non-treatment follow-up phase after completion of the treatment phase, consisting of a clinic visit at week-24 (V11). Modified NUI, eCO levels, blood pressure, heart rate, weight/BMI, and MNWS were recorded in the CRF at this study visit. Collection of saliva samples was repeated.

Saliva samples were collected for cotinine measurement in those who stated they had not vaped and with an eCO ≤ 7 ppm (just to confirm no combustible cigarette use). Participants were asked to chew a small cotton roll (TR0N00RU2, Dentalica, Milano, Italy) for 60 s. Cotton rolls were placed into polypropylene tubes and stored at − 20 °C until use. Cotinine concentrations in saliva samples were analyzed in duplicate by gas chromatography [25]. We adopted a salivary cotinine cut-off for abstinence of 10 ng/ml [26, 27].

### Study outcomes measures

The primary efficacy endpoint of the study was the proportion of subjects with continuous abstinence from vaping between week 4 to week 12 (CAR 4–12 weeks). Abstinence from vaping was defined as cotinine-verified (saliva cotinine < 10 ng/ml) self-reported abstinence from EC use since the last study visit. Secondary efficacy endpoints were CAR 4–24 weeks, and 7-day point prevalence of abstinence at weeks 12, and 24.

Safety endpoints included information on the number of adverse events (AE), and serious adverse events (SAE) occurring between treatment randomization (V1) and the last week of treatment (V10). Between and within treatment groups changes were reported for blood pressure, heart rate, weight, and BMI.

### Trial interventions

#### Vape reduction

A reduction of at least 50% in daily vaping was targeted as a preparatory strategy. Instructions about vaping reduction were specific to the type of vaping product used. Potential study subjects were instructed to gradually taper down daily consumption at their own pace, over time.

#### Cessation medications

Varenicline (0.5 mg tablet) and matched placebo tablets were supplied by the study sponsor randomized and distributed by the hospital pharmacy. Blinding was ensured by the identical appearance of drug and placebo tablets and their containers. Subjects assigned to varenicline were titrated to full dose by the time of their TQD (0.5 mg/day for 2–3 days, 0.5 mg twice daily for 4–5 days; then 1 mg twice daily for 11 weeks), according to the manufacturer’s recommendations.

#### Vaping cessation counseling

Subjects in both treatment groups received the same vaping cessation counseling throughout the whole duration of the study. One-on-one counseling was provided at each visit for a total of 10–15 min by two experienced clinical psychologists. This behavioral intervention is described in detail in the Supplement 2. Briefly, our approach to vaping cessation was partially adapted from the 5A’s brief tobacco interventions for smokers who are ready to quit [24]. *First*, we collected information about participants’ frequency and intensity of use of vaping products (at baseline). *Second*, we *assessed* readiness to quit vaping by asking two questions: “Do you plan to quit vaping within the next 30 days?”, “Do you wish to participate in a vaping cessation program?” Before attending the baseline visit (and committing to a target quit date (TQD)), potential study subjects were asked to reduce the daily use/consumption of their vaping product by at least 50%. When vaping frequency was reduced by 50% (indicating readiness to commit to vaping cessation plan and TQD), they were admitted to the baseline visit. *Third*, those who successfully reduced by 50% their daily use were *assisted* with a quit plan (combining vaping reduction, cessation counseling, use of varenicline, and close follow‑ups). Participants were instructed to set a TQD, ideally within 2 weeks. Participants were reminded of the challenges posed by craving and nicotine withdrawal symptoms when stopping vaping products completely and counseled on how to cope with them to avoid a relapse to vaping (or worse to smoking). Close follow‑up in the first four weeks of the cessation program was arranged to assess participants’ progress, review stress coping skills in order to mitigate the possibility of vaping relapse, address varenicline’s adverse events, and maintain participants’ motivation to quit. Participants were assisted *in dealing with cravings and withdrawal.* As nicotine is an important determinant of e-cigarette dependence, the withdrawal effects experienced upon cessation of vaping may be similar in nature, frequency, and intensity to those experienced when trying to quit tobacco cigarettes. Therefore, vaping cessation counseling may usefully adapt strategies that have long been trained as part of behavioral counseling for smoking cessation. Practical counseling in this study focused on two elements:

1. Helping the participant to identify situations that have historically triggered the individual’s motivation to vape (e.g., social situations, stressful situations, negative emotions).
2. Assisting the participant to practice using a range of cognitive and behavioral coping skills in response to trigger situations.

### Safety reporting

Safety data were summarized for both treatment groups and summary statistics reported. Any events documented in the period from the point of treatment initiation until the last week of treatment (week-12, V10) were considered as relevant to the safety analysis.

Adverse events: all observed or volunteered AEs, regardless of treatment group or suspected causal relationship to the study drug, were recorded. Events involving adverse drug reactions and illnesses with onset during the study were recorded. For all AEs, sufficient information was obtained by the investigator to determine the causality of the AEs.

Serious adverse events: all SAEs (as defined below) regardless of treatment group or suspected relationship to the study drug were reported immediately. A SAE is any adverse drug experience occurring at any dose that (1) results in death, (2) is life-threatening, (3) results in hospitalization or prolongation of existing hospitalization, and (4) results in a persistent or significant disability/incapacity.

### Statistical methods

No success rates on varenicline use among people who exclusively use ECs were available to determine the correct sample size for this study, the first of its type. However, an RCT that involved 139 long-term NRT users to assess the impact of varenicline plus counseling to help people quit NRT revealed a significant difference between the active vs. placebo arm [28]. Consequently, a similar sample size of 140 participants was selected for this study.

Baseline and demographic data are listed for all treatment groups. Summary statistics are reported for each treatment group. At baseline, differences between the varenicline and placebo groups were evaluated by means of one-way analysis of variance (ANOVA) and Mann–Whitney *U* test for normally and non-normally distributed continuous data, respectively; *χ*^2^ test was used to test differences on categorical variables. Secondary endpoints were analyzed using procedures similar to that described above for the primary endpoint. Intention-to-treat analyses were adopted for efficacy evaluation, on the assumption that subjects lost to follow-up continued vaping.

Safety data were summarized for both treatment groups and summary statistics were reported. Any events documented in the period from the point of treatment initiation until the last week of treatment (week-12, V10) were considered as relevant to the safety analysis.

Aimed at identifying predictors of continuous vaping abstinence, a multiple logistic regression model was estimated in which “continuous abstinence between weeks 4 and 12” (yes/no) was entered as the criterion variable. Putative predictors were selected by a priori evaluation among a series of baseline characteristics, which were entered in the model as covariates and included: age, gender, education level, years of smoking prior to regular vaping, years of exclusive daily vaping, number of quit vaping attempts, motivation levels by VAS, cohabitant vapers, BDI II score, BAI score, PSECDI score, MNWS at week-4, weight increase at week-12, and study group. The analysis was also repeated considering as covariates those baseline characteristics that were found to be significantly different between study groups (as per Table 1).

**Table 1:** Baseline characteristics of study participants by treatment group

Characteristic	Varenicline group (*N* = 70) Mean (± SD)	Placebo group (*N* = 70) Mean (± SD)	*p*-value
Age (years)	53.8 (9.7)	51.3 (8.4)	*0.0196*
Years of smoking^a^	27.7 (7.4)	27.1 (7.3)	*0.6484*
Years of vaping^b^	2.0 (1.4)	2.1 (1.00)	*0.1714*
Motivation level by VAS	8.0 (7–10)^c^	8.5 (7–10)^c^	*0.9611*
BDI	7 (3–12.8)^c^	9 (5–14)^c^	*0.2895*
BAI	6 (3–13.5)^c^	9.5 (5–17)^c^	*0.0151*
MNWS^**d**^	9 (7–12)^c^	9 (6–12)^c^	*0.8413*
PSECDI	11.7 (6.2)	14.9 (7.3)	*0.0283*
Weight (kg)	75.2 (12.6)	79.1 (15.8)	*0.1095*
Height (cm)	167.8 (8.4)	168.6 (9.5)	*0.5891*
BMI	26.95 (4.20)	27.63 (4.04)	*0.3289*
SBP (mmHg)	126.9 (13.3)	127.6 (13.3)	*0.7366*
DBP (mmHg)	76.4 (8.1)	77.4 (10.0)	*0.5092*
HR (b/min)	74.4 (10.2)	77.2 (10.4)	*0.1066*
	Varenicline group (*N* = 70) No. (%)	Placebo group (*N* = 70) No. (%)	*p*-value
Gender			*0.8657*
M	36 (51.4%)	33 (47.1%)	
F	34 (48.6%)	37 (52.9%)	
Marital status			*0.7046*
Married	53 (75.7%)	55 (78.6%)	
Unmarried	7 (10.0%)	8 (11.4%)	
Divorced	5 (7.1%)	1 (1.4%)	
Widower	2 (2.9%)	3 (4.3%)	
Separated	2 (2.9%)	2 (2.9%)	
Cohabiting	1 (1.4%)	1 (1.4%)	
Educational level			*0.0415*
No education	0 (0%)	2 (2.8%)	
Elementary school	6 (8.6%)	8 (11.4%)	
Middle school	25 (35.7%)	30 (42.9%)	
High school	25 (35.7%)	27 (38.6%)	
Graduation	14 (20.0%)	3 (4.3%)	
Cohabitant vapers			*0.3357*
YES	32 (45.7%)	30 (42.9%)	
NO	38 (54.3%)	40 (57.1%)	
Number of quit vaping attempts			*0.2355*
> 1	41 (58.6%)	34 (48.6%)	
1	29 (41.4%)	36 (51.4%)	
Main vaping device^e^			*0.7107*
Refillable tank	55 (78.6%)	53 (75.7%)	
Refillable pod/cartridge	9 (12.9%)	10 (14.3%)	
Closed pod/cartridge system	5 (7.1%)	7 (10.0%)	
Disposable	1 (1.4%)	0 (0.0%)	
Main e-liquid flavor^**f**^			*0.6909*
Tobacco	32 (45.7%)	34 (48.5%)	
Fruit	15 (21.4%)	17 (24.3%)	
Mint	6 (8.6%)	4 (5.7%)	
Dessert	11 (15.7%)	9 (12.9%)	
Mixed	6 (8.6%)	6 (8.6%)	

^a^ Previous smoking years, prior to regular vaping. All EC users in the study were former smokers

^b^ Years of exclusive vaping. All EC users in the study were vaping daily

^c^ Median (IQR)

^d^ MNWS, measured at week-4 (varenicline, *n* = 58; placebo, *n* = 56)

^e^ A secondary device was used (15.7% and 18.6% of cases — in placebo and varenicline groups, respectively)

^f^ Alternative e-liquids were often used (45.7% and 47.1% of cases — in placebo and varenicline groups, respectively)

## Results

### Trial participants

The first 140 consecutive eligible subjects were randomized to receive the active drug or placebo. One hundred and thirteen participants completed all the visits within the treatment phase, of whom 57 were in the varenicline group and 56 in the placebo group Fig. 1. The 24-week study visit (nontreatment phase) was completed by 95 subjects, of whom 51 were in the varenicline group and 44 were in the placebo group. Subjects’ baseline characteristics between groups were comparable with the exception of age, BAI, PSECDI, and educational level (Table 1). Subjects had a mean (SD) age of 52.6 (9.1) years, and smoked 15–20 cigarettes daily for at least 25 years before switching to vaping products. Participants were adults who have been using ECs daily for at least 2 years, had made at least one serious quit vaping attempt in the past, and had a mean (SD) PSECDI score of 11.7 (6.2) for the varenicline group and 14.9 (7.3) for the placebo group, indicating a high level of EC dependency. Participants self-reported reduction in daily EC use by at least 50%, and had a VAS motivation score > 8, indicating strong motivation to quit vaping. The reasons most commonly endorsed for an interest in quitting vaping were: a) concern about the potential health risks of long-term EC use (72.9%); b) desire to break dependence on vaping products (62.1%); c) the experience of some adverse physical effects (e.g., dry mouth, sore throat, dry cough) (27.9%); and d) the increasing cost of vaping (23.6%).

### Vaping abstinence rates

**Fig. 3:**
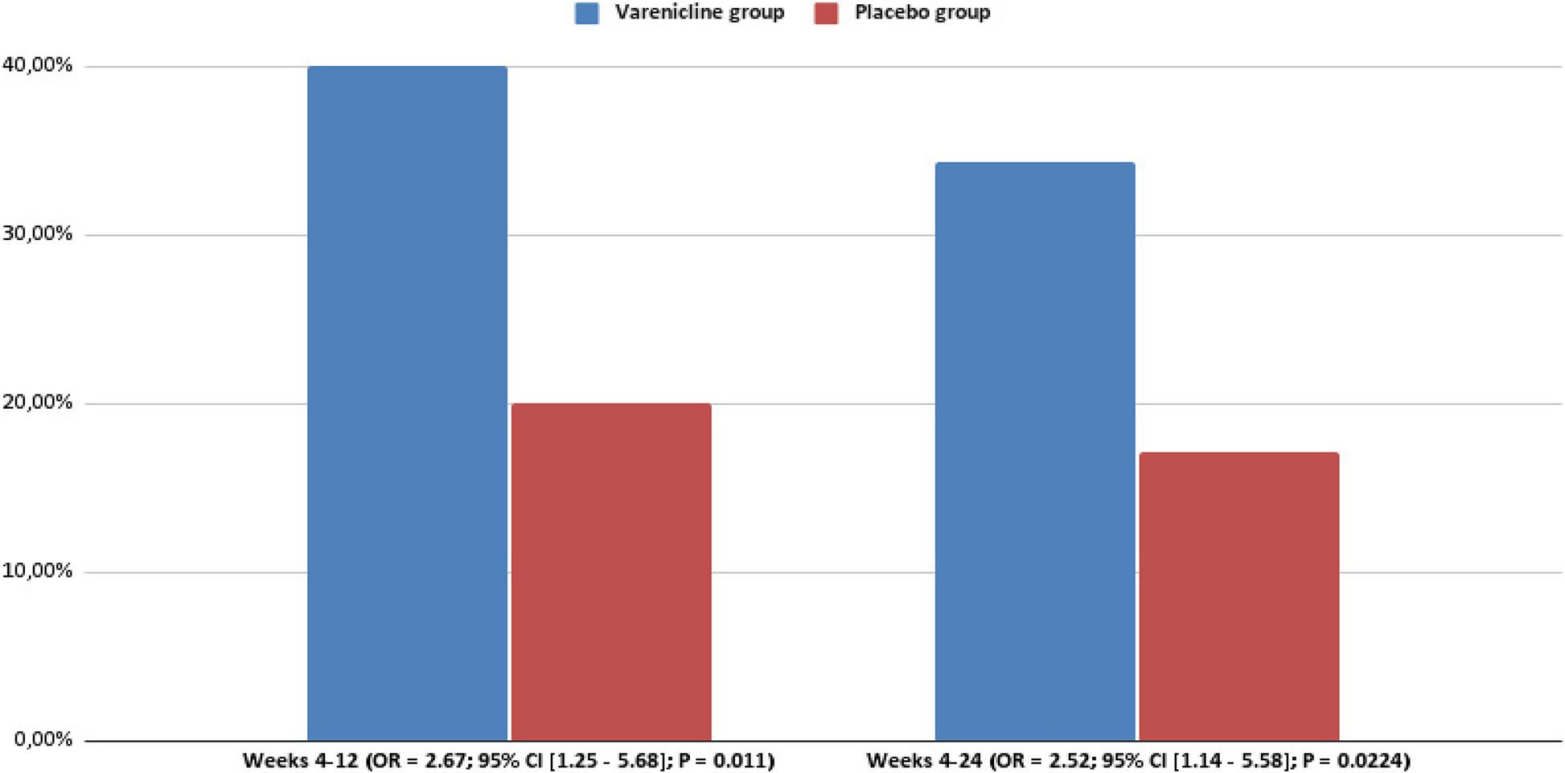
Efficacy CAR. Proportion of participants who reported abstinence from vaping, which was defined by cotinine level-verified self-reported abstinence. Primary efficacy end point was the continuous abstinence rate at weeks 4 to 12

The cotinine level-verified CARs for weeks 4–12 and weeks 4–24 are shown in Fig. 3 and Table 2. CARs were significantly higher for varenicline vs placebo at each interval: weeks 4–12, 40.0% and 20.0%, respectively (OR = 2.67, 95% CI = [1.25–5.68], *P* = 0.011); weeks 4–24, 34.3% for varenicline and 17.2% for placebo (OR = 2.52, 95% CI = [1.14–5.58], *P* = 0.0224).

**Table 2:** Continuous abstinence rates and 7-day point prevalence

	**Varenicline group**	**Placebo group**	**OR**	**95% CI**	***p*-value**
**Continuous abstinence rate**					
CAR 4–12 weeks	40.0%	20.0%	2.67	[1.25–5.68]	***0.011***
CAR 4–24 weeks	34.3%	17.1%	2.52	[1.14–5.58]	***0.022***
**7-day point prevalence**					
Week-4	41.4%	22.9%	2.39	[1.15–4.97]	***0.020***
Week-5	41.4%	27.1%	1.86	[0.93–3.86]	*0.076*
Week-6	40.0%	25.7%	1.93	[0.94–3.95]	*0.073*
Week-7	40.0%	25.7%	1.93	[0.94–3.95]	*0.073*
Week-8	40.0%	20.0%	2.67	[1.25–5.68]	***0.011***
Week-12	40.0%	20.0%	2.67	[1.25–5.68]	***0.011***
Week-24	34.3%	17.1%	2.52	[1.14–5.58]	***0.022***

The 7-day point prevalence of vaping abstinence was also higher for the varenicline than placebo at each time point (Table 2); in particular, significant results were shown at week 4, 41.4% vs 22.9% (OR = 2.39, 95% CI = [1.15–4.97], *P* = 0.02; week 12, 40.0% vs 20.0% (OR = 2.67, 95% CI = [1.25–5.68], *P* = 0.011); and week 24, 34.3 vs 17.2 (OR = 2.52, 95% CI = [1.14–5.58], *P* = 0.0224).

Of note, none of the factors that were found to be significantly different at baseline (i.e. age, BAI, PSECDI, and education level) contributed to the effect of varenicline on CAR for weeks 4–12 (see Additional file 1: Table S2). The use of varenicline remained significantly predictive of cessation after controlling for the effects of these variables in the model ( Additional file 1: Table S2).

### Changes in vaping behavior

EC users who were not abstinent from vaping were considered as treatment failures. Taking the whole cohort of subjects completing the study, reduction in vaping consumption was observed in 33.6% and 26.3% of the subjects at week 12 and week 24, respectively. Reduction in vaping consumption between the groups was significant (*P* = 0.002, at week 12; *P* = 0.0289, at week 24) **(**see Additional file 1: Table S3).

Vaping relapse was observed in 29.2% and 35.8% of the subjects at week 12 (V6) and week 24 (V7), respectively. Vaping relapse rate during the non-treatment phase was calculated by considering changes in behavior trajectories from V6 to V7 ( Additional file 1: Table S3). For the *intention-to-treat* analysis, calculations considered variations in the number of relapsing vapers added to the increase in the number of LTFUs (as it is presumed that LTFUs have relapsed back into vaping in the intention-to-treat analysis) and then divided by the total number of participants. We observed an increase in vaping relapse rate after drug withdrawal in the varenicline group vs. the placebo group (+ 17.2% vs. + 9.9%). For the *per protocol* analysis, calculations considered variations in the number of relapsing vapers divided by the number of participants attending the study visit. A marked increase in vaping relapse rate was noted during the non-treatment phase in the varenicline group vs. the placebo group in the per protocol analysis (+ 13.0% vs. + 1.8%). No subject in the study relapsed to tobacco cigarette smoking. Details of changes in vaping status at each study visit are illustrated in Additional file 1: Table S3.

### Clinical and demographic features influencing vaping abstinence

A multiple logistic regression model estimated the varenicline group vs the placebo group had an OR of 3.2 (95% CI, 1.19–8.60; *P* = 0.021) for the CAR at weeks 4 to 12 ( Additional file 1: Table S4). Having cohabitant vapers reduced the odds of success for CAR by approx. 70% (OR, 0.284; 95% CI, 0.091–0.888; *P* = 0.030). BAI scores were also associated with reduced odds of success for CAR (OR, 0.219; 95% CI, 0.055–0.870; *P* = 0.031). Non-significant trends were observed for PSECDI (*p* = 0.054) and craving score (*p* = 0.059).

### Adverse events

Total number of AEs was significantly greater in the varenicline group than in the placebo group (246 vs. 154; *P* = 0.042). However, most AEs were rated as mild or moderate and rarely led to treatment discontinuation; two in the varenicline group and one in the placebo group. The AEs that occurred more frequently in the varenicline group than in the placebo group were nausea (49 [19.9%] vs 19 [12.3%]), flatulence (17 [6.9%] vs 6 [3.9%]), and abnormal dreams (16 [6.5%] vs 5 [3.3%]) (Additional file 1: Table S5).

No significant changes in mean (SD) systolic and diastolic blood pressure, and resting heart rate were observed between and within treatment groups ( Additional file 1: Table S6 A, B). Weight gain was observed among subjects in the varenicline group; however, the increase of 1.4 kg and 2.2 kg at week-12 and week-24 was non-significant. The frequency of most commonly reported respiratory/oral AEs (such as dry mouth, sore throat, cough) was reduced by the end of the study, lower in the varenicline compared with the placebo group.

Measures of the urge to vape across the treatment phase of this study were consistently attenuated with varenicline; at week-4, MNWS craving sub-score of 0.56 (0–2) in the varenicline group was significantly lower than 1.61 (0–2.75) (*P* = 0.0018) in the placebo group.

## Discussion

In our experience and according to recent surveys both youth and adults demonstrate a growing interest in quitting ECs [29–31]. In this study, participants’ strong desire to quit vaping was largely due to concern about the potential health risks of long-term EC use and the need to break the dependency on vaping products. In spite of the growing interest in vaping cessation, there is no evidence to inform recommendations to assist adults who use ECs intending to stop using their vaping products [32]. This RCT is the first to investigate the efficacy and safety of FDA-approved smoking cessation medications for aiding vaping cessation in adults who use ECs. The findings suggest that varenicline can help them to give up vaping and has an acceptable safety profile.

Varenicline was consistently superior to placebo at week-12 and week-24. Among people who use ECs, varenicline more than doubled the chance to quit vaping compared with placebo; cotinine level–verified CAR in the varenicline group combined with vaping cessation counseling was 40.0% at weeks 4 to 12, and 34.3% at weeks 4 to 24. These results are quite remarkable considering that varenicline only alleviates nicotine withdrawal symptoms and cravings, but cannot replace the need for vaping-related rituals. The ORs for the varenicline group in the present trial were similar than the ORs in previous RCTs of smoking cessation [33, 34], NRT cessation [28], and smokeless tobacco cessation [35]. These findings suggest that EC users with a relatively high EC dependency (as shown by their previous history of failed quit attempts and relatively high PSECDI score) have similar difficulty quitting compared with smokers in the general population, yet a number do respond to the intervention.

Varenicline is a specific partial agonist and antagonist of the α4β2 nicotinic acetylcholine receptor that has been found to be effective in increasing abstinence rates among cigarette smokers. It is expected to help with the cessation of nicotine vaping in light of its mechanism of action that attenuates nicotine withdrawal symptoms and craving [36, 37]. In line with this observation in cigarette smokers, varenicline was shown to be consistently effective at reducing the urge to vape in people who use ECs. Another mechanism by which varenicline facilitates sustained abstinence is by reducing the likelihood of relapse to smoking during a quit attempt [33, 34, 38]. Although relapse prevention was not formally investigated, this effect of varenicline was confirmed in the present study; after stopping varenicline (between week-12 and week-24) vaping relapse rate increased by 17.2% compared to 9.9% after stopping placebo.

Presence of cohabitant vapers and high level of anxiety greatly reduced the odds of success for abstinence from vaping, similar to what is observed in cigarette smokers [39]. Smokers with anxiety disorders have more severe withdrawal symptoms during smoking cessation than smokers without anxiety disorders and are less likely to quit [40]. As for people who smoke, we found that high levels of anxiety were significantly associated with reduced odds of sustained vaping cessation in EC users. The presence of smokers in the household is known to be among the strongest sociodemographic predictors of quitting smoking in adult cigarette smokers [41, 42]. In the future, cessation interventions for EC users should take into consideration these specific modifiers.

The safety profile of varenicline in this study was good and similar to that of previous varenicline trials of smokers in the general population [33, 34]. Nausea, flatulence, and abnormal dreams occurred more frequently in the varenicline group than in the placebo group. As a consequence of vaping cessation, the frequency of dry mouth, sore throat, and cough was reduced by the end of the study and much lower in the varenicline compared with the placebo group. No difference in blood pressure and heart rate was noted throughout the intervention. A gradual gain in weight was observed in the varenicline group, but the increase was non-significant compared to placebo.

This RCT has several strengths: (1) use of continuous abstinence rate as a robust primary efficacy endpoint of the study; (2) use of salivary cotinine measurements to objectively verify abstinence from vaping; (3) careful verification of compliance with study medications attained by drug adherence checks; (4) detailed characterization of study participants, that include their vaping patterns, details of their vaping products, and their reasons to quit vaping; (5) use of specific vaping cessation counseling and vaping tapering plan for the study.

Despite these strengths, the study has several limitations. First, findings in a population of adults who use ECs cannot be extended to young users. Nonetheless, the potential of behavioral interventions for vaping cessation in vapers 18–24 years old has been recently reported [43]. Second, findings were restricted to a selected population of participants who had a strong desire to quit ECs, high EC dependency, and used almost exclusively refillable vaping products, thus limiting the generalizability of the results. Third, the 6-month follow-up is limited and longer follow-up should be considered in future studies. Fourth, the impact of vaping cessation counseling could not be assessed as the study was not designed to test the isolated effect of the behavioral intervention. Lastly, groups were not well-matched for anxiety and EC dependence level. It is possible that the significantly higher level of anxiety and EC dependence in the placebo group at baseline might have contributed to attenuate success rates in this study group. Nonetheless, our multivariate logistic regression model showed that varenicline remained significantly predictive of cessation after controlling for the effects of anxiety and EC dependence level, suggesting that varenicline's therapeutic efficacy is not limited to subsets of vapers.

## Conclusions

Vaping cessation is virtually unexplored and there is a clear need for treatment protocols and guidelines to advance best practices and outcomes for people who use ECs and want to quit. In particular, the efficacy and safety of medications approved for smoking cessation by the U.S. FDA for aiding vaping cessation have never been investigated. The findings of the present RCT indicate that the inclusion of varenicline in a vaping cessation program for adults who use ECs and intend to quit may result in prolonged abstinence without serious adverse events. This evidence supports the use of varenicline in cessation programs to help EC users stop vaping and may inform future recommendations by health authorities and healthcare providers. Studies with longer follow-ups should be conducted to evaluate long-term efficacy.

## Supplementary Information

**Additional file 1: Table S1.** Study Schedule/Assessments. **Table S2.** Multivariate Logistic Regression Model for CAR 4–12 Week considering each factor significantly different at baseline as covariates. **Table S3.** Change in vaping behavior throughout the study. **Table S4.** Multivariate Logistic Regression Model for CAR 4–12 Week. **Table S5.** Summary of adverse events occurring in > 3% of either treatment group. **Table S6A, B.** Metabolic and Cardiovascular Parameters Measured at Baseline and Week 12, Metabolic and Cardiovascular Parameters Measured at Baseline and Week 12.

**Additional file 2.**

## Data Availability

The datasets used and analyzed during the current study are available from the corresponding author on reasonable request.

## Abbreviations

AEs: Adverse events
ANOVA: Analysis of variance
BAI: Beck Anxiety Inventory
BDI-II: Beck Depression Inventory-II
BMI: Body mass index
CAR: Continuous abstinence from vaping
CAR: Continuous abstinence rate
CI: Confidence interval
CoEHAR: Center of Excellence for the Acceleration of HArm Reduction
CONSORT: Consolidated Standards of Reporting Trials
CPCT: Centre for the Prevention and Treatment of Tobacco Addiction
EC: Electronic cigarette
eCO: Exhaled carbon monoxide
EudraCT: European Union Drug Regulating Authorities Clinical Trials Database
FDA: Food and Drug Administration
INNCO: International Network of Nicotine Consumers Organizations
LIAF: Italian Anti-Smoking League
mcEq: Modified cigarette evaluation questionnaire
Mnui: Modified Nicotine Use Inventory
MNWS: Minnesota Nicotine Withdrawal Scale
OR: Odds ratio
P: Observed level of significance
PSECDI: Penn State Electronic Cigarette Dependence Index
RCT: Randomized controlled trial
TQD: Target quit date
UOC MCAU: Complex operating unit of Medicine and Surgery of Acceptance and Urgency
V1: Baseline visit
VAS: Visual analog scale
