# Supplementary material for: Varenicline and counseling for vaping cessation: a double-blind, randomized, parallel-group, placebo-controlled trial
Source: BMC Med. 2023 Jul 5;21:220. doi: 10.1186/s12916-023-02919-2 (PMC10321010; doi:10.1186/s12916-023-02919-2)
Supplement: Supplementary file 1 — Additional file 1: Table S1. Study Schedule/Assessments. Table S2. Multivariate Logistic Regression Model for CAR 4–12 Week considering each factor significantly different at baseline as covariates. Table S3. Change in vaping behavior throughout the study. Table S4. Multivariate Logistic Regression Model for CAR 4–12 Week. Table S5. Summary of adverse events occurring in > 3% of either treatment group. Table S6A, B. Metabolic and Cardiovascular Parameters Measured at Baseline and Week 12, Metabolic and Cardiovascular Parameters Measured at Baseline and Week 12. [file 12916_2023_2919_MOESM1_ESM.docx]

ADDITIONAL FILE

Table S1 Study Schedule/Assessments

Table S2 - Multivariate Logistic Regression Model for CAR 4-12 Week considering each factor significantly different at baseline as covariates

Table S3 – Change in vaping behavior throughout the study

Table S4 - Multivariate Logistic Regression Model for CAR 4-12 Week

Table S5. Summary of adverse events occurring in > 3% of either treatment group

Table S6A - Metabolic and Cardiovascular Parameters Measured at Baseline and Week 12

TABLE S6B - Metabolic and Cardiovascular Parameters Measured at Baseline and Week 24

**Table S1.** **Study Schedule/Assessments**

| **Procedure** | **Screen** | **BL** | **Wk**  **1** | **Wk**  **2** | **Wk 3** | **Wk**  **4** | **Wk 5** | **Wk**  **6** | **Wk 7** | **Wk**  **8** | **Wk**  **12** | **Wk**  **24** |
| --- | --- | --- | --- | --- | --- | --- | --- | --- | --- | --- | --- | --- |
|  | **VISIT**  **0** | **VISIT 1** | **VISIT 2** | **VISIT 3** | **TC** | **VISIT 5** | **TC** | **VISIT 7** | **TC** | **VISIT 9** | **VISIT 10** | **VISIT 11** |
| Eligibility criteria | **X** | **X** |  |  |  |  |  |  |  |  |  |  |
| Informed consent |  | **X** |  |  |  |  |  |  |  |  |  |  |
| Smoking and vaping history | **X** | **X** |  |  |  |  |  |  |  |  |  |  |
| HR, BP |  | **X** | **X** | **X** |  | **X** |  | **X** |  | **X** | **X** | **X** |
| Weight/BMI |  | **X** |  |  |  |  |  |  |  |  | **X** | **X** |
| Socio-demographic factors |  | **X** |  |  |  |  |  |  |  |  |  |  |
| Adverse events |  | **X** | **X** | **X** | **X** | **X** | **X** | **X** | **X** | **X** | **X** |  |
| Motivation by VAS |  | **X** |  |  |  |  |  |  |  |  |  |  |
| BDI & BAI |  | **X** |  |  |  |  |  |  |  |  |  |  |
| MNWS |  |  | **X** | **X** |  | **X** |  | **X** |  | **X** | **X** | **X** |
| PSECDI |  | **X** |  |  |  |  |  |  |  |  |  |  |
| mNUI |  | **X** | **X** | **X** | **X** | **X** | **X** | **X** | **X** | **X** | **X** | **X** |
| eCO |  | **X** | **X** | **X** |  | **X** |  | **X** |  | **X** | **X** | **X** |
| Saliva cotinine* |  |  |  |  |  | **X** |  | **X** |  | **X** | **X** | **X** |
| Counselling |  | **X** | **X** | **X** | **X** | **X** | **X** | **X** | **X** | **X** | **X** | **X** |
| Dispense study drug |  | **X** | **X** | **X** |  | **X** |  | **X** |  | **X** |  |  |
| Dosing record |  |  | **X** | **X** |  | **X** |  | **X** |  | **X** | **X** |  |

BL=baseline; BDI=Beck Depression Inventory; BAI=Beck Anxiety Inventory; eCO=exhaled carbon monoxide; PSECDI: Penn State Electronic Cigarette Dependence Index; mNUI: modified Nicotine Use Inventory; HR=heart rate; BP=blood pressure; BMI= Body Mass Index; MNWS: Minnesota Nicotine Withdrawal Scale: TC=telephone contact; VAS=visual analog score

*Vaping cessation was objectively verified by saliva cotinine measurements in those who self-reported complete abstinence from EC use at weeks 4, 6, 8, 12, and 24.

**TABLE S2 Multivariate Logistic Regression Model for CAR 4-12 Week considering each factor significantly different at baseline against Varenicline vs. Placebo**

|  | **OR** | **(95% CI) Lower** | **(95% CI) Upper** | **p** |
| --- | --- | --- | --- | --- |
| **Age** | 0.985 | 0.944 | 1.03 | 0.471 |
| **BAI score** | 0.983 | 0.938 | 1.03 | 0.485 |
| **PSECDI score** | 0.977 | 0.899 | 1.06 | 0.588 |
| **Educatiol level** | 1.085 | 0.67 | 1.76 | 0.740 |
| **Varenicline vs. Placebo** | 2.454 | 1.035 | 5.82 | **0.042** |

**TABLE S3 – Change in vaping behavior* throughout the study**

|  | **Placebo**  **N = 70** |  |  |  |  | **Varenicline**  **N = 70** |  |  |  |  |  |
| --- | --- | --- | --- | --- | --- | --- | --- | --- | --- | --- | --- |
|  | Total Subjects N | LTFU N | Vape Reducers N | Vape Relapsers N | Vape Quitters N (%) | Total Subjects N | LTFU N | Vape Reducers N | Vape Relapsers N | Vape Quitters N (%) | p-value |
| At week-4 | 58 | 12 | 17 | 25 | 16 (22.9%) | 58 | 12 | 19 | 10 | 29 (41.4%) | **0.0162** |
| At week-5 | 58 | 12 | 15 | 24 | 19 (27.1%) | 57 | 13 | 17 | 11 | 29 (41.4%) | 0.0695 |
| At week-6 | 58 | 12 | 15 | 25 | 18 (25.7%) | 57 | 13 | 19 | 10 | 28 (40.0%) | **0.0278** |
| At weeks-7 | 58 | 12 | 16 | 24 | 18 (25.7%) | 57 | 13 | 21 | 8 | 28 (40.0%) | **0.0123** |
| At weeks-8 | 56 | 14 | 17 | 25 | 14 (20.0%) | 57 | 13 | 22 | 7 | 28 (40.0%) | **0.0015** |
| At week-12 | 56 | 14 | 15 | 27 | 14 (20.0%) | 57 | 13 | 23 | 6 | 28 (40.0%) | **0.0002** |
| At week-24 | 44 | 26 | 10 | 22 | 12 (17.1%) | 51 | 19 | 15 | 12 | 24 (34.3%) | **0.0289** |

**self-reported (cotinine-verified at 4-, 6-, 8-, 12-, and 24-weeks)*

*LTFU – lost to follow up*

**TABLE S4 - Multivariate Logistic Regression Model for CAR 4-12 Week**

|  | **OR** | **(95% CI) Lower** | **(95% CI) Upper** | **p** |
| --- | --- | --- | --- | --- |
| **Age** | 0.975 | 0.906 | 1.049 | 0.492 |
| **Gender** | 0.299 | 0.085 | 1.053 | 0.060 |
| **Years of smoking*** | 0.997 | 0.906 | 1.098 | 0.954 |
| **Years of vaping**** | 1.026 | 0.628 | 1.677 | 0.919 |
| **Cohabitant vapers** | 0.284 | 0.091 | 0.888 | **0.030** |
| **Previous quit vaping attempts** | 1.872 | 0.585 | 5.993 | 0.291 |
| **BDI** | 2.038 | 0.353 | 9.766 | 0.426 |
| **BAI** | 0.219 | 0.055 | 0.870 | **0.031** |
| **PSECDI** | 0.337 | 0.111 | 1.019 | 0.054 |
| **Motivation level by VAS** | 1.077 | 0.830 | 1.397 | 0.579 |
| **MNWS^$^**  Total score  Craving score | 2.206  0.357 | 0.602  0.115 | 8.080  1.099 | 0.232  0.059 |
| **Weight increase at week-12** | 1.093 | 0.877 | 1.364 | 0.429 |
| **Varenicline vs. Placebo** | 3.196 | 1.188 | 8.599 | **0.021** |

* Previous smoking years, prior to regular vaping. All EC users in the study were former smokers

** Years of exclusive vaping. All EC users in the study were vaping daily

^$^ MNWS, measured at week-4 (varenicline, n = 58; placebo, n = 56)

**Table S5. Summary of adverse events occurring in ³ 3% of either treatment group**

|  | *Varenicline*  *n(%)* | *Placebo*  *n(%)* | *p-value* |
| --- | --- | --- | --- |
| *Adverse Events**  *Total reported events* | *(N = 246)* | *(N = 154)* | *0.042* |
| *Nausea* | *49 (19.9%)* | *19 (12.3%)* |  |
| *Headache/Migraine* | *25 (10.2%)* | *16 (10.4%)* |  |
| *Dry mouth* | *24 ( 9.8%)* | *22 (14.3%)* |  |
| *Insomnia* | *27 (11.0%)* | *12 ( 7.8%)* |  |
| *Flatulence* | *17 ( 6.9%)* | *6 ( 3.9%)* |  |
| *Abnormal dreams* | *16 ( 6.5%)* | *5 ( 3.3%)* |  |
| *Anxiety* | *16 ( 6.5%)* | *9 ( 5.8%)* |  |
| *Irritability* | *14 ( 5.7%)* | *10 ( 6.5%)* |  |
| *Sore throat* | *16 ( 6.5%)* | *16 (10.4%)* |  |
| *Constipation* | *10 ( 4.1%)* | *9 ( 5.8%)* |  |
| *Cough* | *10 ( 4.1%)* | *9 ( 5.8%)* |  |
| *Lightheadedness/dizziness* | *10 ( 4.1%)* | *8 ( 5.2%)* |  |
| *Others* | *12 ( 4.9%)* | *13 ( 8.4%)* |  |

**Adverse events recorded during treatment phase and occurring in 3% or more of either treatment group. Participants were counted only once per row but could be counted several times per column.*

**TABLE S6A - Metabolic and Cardiovascular Parameters Measured at Baseline and Week 12**

|  | **Baseline**  **mean (SD)** | | **Week 12**  **mean (SD)** | | *P value*  *Δ* | *P value*  *Φ* | *P value*  *Ψ* |
| --- | --- | --- | --- | --- | --- | --- | --- |
|  | **Varenicline** | **Placebo** | **Varenicline** | **Placebo** |  |  |  |
| **Weight (Kg)** | 75.5 (13.4) | 78.4 (15.2) | 76.9 (14.2) | 78.8 (15.1) | *0.0742* | *0.9315* | *0.0965* |
| **BMI** | 26.81 (4.52) | 27.76 (4.61) | 27.12 (4.52) | 27.88 (4.03) | *0.1175* | *0.8724* | *0.3434* |
| **SBP (mmHg)** | 126.2 (11.8) | 128.0 (12.7) | 125.1 (9.6) | 126.4 (8.9) | *0.6175* | *0.0864* | *0.8834* |
| **DBP (mmHg)** | 76.1 (8.1) | 77.8 (10.5) | 76.7 (8.6) | 77.20 (9.4) | *0.0904* | *0.7602* | *0.3404* |
| **HR (b/m)** | 74.7 (10.4) | 76.7 (10.8) | 73.8 (8.7) | 75.4 (11.6) | *0.6282* | *0.5288* | *0.3979* |

*Δ =* Baseline vs. Week12 – Varenicline (N=57)

*Φ =* Baseline vs. Week12 – Placebo (N=56)

*Ψ =* Varenicline vs. Placebo

**TABLE S6B - Metabolic and Cardiovascular Parameters Measured at Baseline and Week 24**

|  | **Baseline**  **mean (SD)** | | **Week 24**  **mean (SD)** | | *P value*  *Δ* | *P value*  *Φ* | *P value*  *Ψ* |
| --- | --- | --- | --- | --- | --- | --- | --- |
|  | **Varenicline** | **Placebo** | **Varenicline** | **Placebo** |  |  |  |
| **Weight (Kg)** | 74.6 (13.7) | 77.8 (14.2) | 76.8 (14.5) | 78.3 (14.1) | *0.0643* | *0.9415* | *0.0923* |
| **BMI** | 26.56 (4.65) | 27.84 (4.02) | 26.98 (4.56) | 27.93 (4.00) | *0.6468* | *0.9212* | *0.2857* |
| **SBP (mmHg)** | 125.4 (12.1) | 128.2 (12.6) | 124.4 (8.3) | 126.0 (8.2) | *0.6218* | *0.0549* | *0.7022* |
| **DBP (mmHg)** | 76.2 (8.2) | 76.8 (10.1) | 78.0 (8.9) | 77.2 (8.4) | *0.1075* | *0.8451* | *0.3453* |
| **HR (b/m)** | 75.1 (10.4) | 78.2 (11.3) | 74.5 (8.9) | 76.7 (12.2) | *0.7134* | *0.3181* | *0.5787* |

*Δ =* Baseline vs. Week24 – Varenicline (N=51)

*Φ =* Baseline vs. Week24 – Placebo (N=44)

*Ψ =* Varenicline vs. Placebo
