# Supplementary material for: Varenicline and counseling for vaping cessation: a double-blind, randomized, parallel-group, placebo-controlled trial
Source: BMC Med. 2023 Jul 5;21:220. doi: 10.1186/s12916-023-02919-2 (PMC10321010; doi:10.1186/s12916-023-02919-2)
Supplement: Supplementary file 2 — Additional file 2. [file 12916_2023_2919_MOESM2_ESM.docx]

**SUPPLEMENT 2**

**Vaping cessation counselling**

Subjects in both treatment groups received the same vaping cessation counselling throughout the whole duration of the study. One-on-one counselling were provided at each visit for a total of 10-15 minutes. Two experienced clinical psychologists delivered this counselling. Our approach to vaping cessation was partially adapted from the 5A’s brief tobacco interventions for smokers who are ready to quit (**i**). This behavioral intervention is described in detail below.

First, we collected information about participants’ frequency and intensity of use of vaping products (at baseline) by **asking**:

“Would mind if I take a look at your vape/e-cigarette?”

“How often do you vape in a day?”

“How much do you normally vape?”

*Depending on the device, questions were modified as:*

*“On average, how many cartridge/pods did you consume yesterday/last week/last month?”*

*“On average, how much e-liquid did you consume yesterday/last week/last month (in ml)?”*

*“What level of nicotine do you use in your vaping product (in mg/ml)?”*

“How soon after you wake up do you first use your vape (i.e. time to first vape)?”

“When is the last time you vaped?”

“Why have you decided to quit vaping?” or “What made you decide to quit vaping?”

Second, we **assessed** readiness to quit vaping by asking two questions: ‘‘Do you plan to quit vaping within the next 30 days?’’, ‘‘Do you wish to participate in a vaping cessation program?’’ Before attending the baseline visit (and committing to a target quit date - TQD), potential study subjects were asked to reduce the daily use/consumption of their vaping product by at least 50%. Depending on the type of vaping product used, we used different definitions to indicate a 50% reduction. If the users was vaping a refilling device, the 50% reduction is indicated by a reduction in e-liquid volume consumed per day (e.g. reducing down to at least 2 ml/day if he/she vaped on average 4 ml/day). If the users was vaping a closed system (e.g. prefilled pod/cartridge) the 50% reduction is indicated by doubling the no. days over which the same volume in consumed (e.g. reducing down to at least 1 pod every two days if he/she consumed 1pod/day). Potential study subjects were instructed to gradually taper down daily consumption at their own pace, over time. When vaping frequency was reduced by 50% (indicating readiness to commit to vaping cessation plan and TQD), they were admitted to the baseline visit.

Third, those who successfully reduced by 50% their daily use were **assisted** with a quit plan (combining vaping reduction, cessation counseling, use of varenicline, and close follow‑ups). Participants were instructed to set a TQD, ideally within two weeks. This is an important step, but one on which participants must take the lead and choose for themselves because setting a TQD creates accountability and indicates serious commitment. Participants were instructed to increase varenicline dosing prior to the chosen TQD according to manufacturer’s recommendations. Varenicline treatment lasted for 12 weeks. Participants were reminded of the challenges posed by craving and nicotine withdrawal symptoms when stopping vaping products completely and counseled on how to cope with them to avoid a relapse to vaping (or worse to smoking). Close follow‑up in the first four weeks of the cessation program was arranged to assess participants’ progress, review stress coping skills in order to mitigate the possibility of vaping relapse, address varenicline’s adverse events, and maintain participants’ motivation to quit.

Participants were motivated to stop vaping and therefore in a stage of *determination* to change. As a consequence, our counseling activity aimed to facilitate participants’ transition to the stage of *action* and to keep the desired change when reached (**ii**). This is normally achieved by assisting in dealing with cravings and withdrawal symptoms, and by promptly addressing slips and relapses.

***Assisting in dealing with cravings and withdrawal symptoms***

Although there is no formal guidance on how to quit vaping, there is a wealth of information on how to cope with the nicotine withdrawal syndrome that reliably follows cessation of cigarette smoking. As nicotine is an important determinant of e-cigarette dependence, the withdrawal effects experienced upon cessation of vaping may be similar in nature, frequency, and intensity to those experienced when trying to quit tobacco cigarettes. One of the biggest challenges for those who try to quit smoking or vaping is coping with cravings and other nicotine withdrawal effects (**iii, iv, v**). Therefore, vaping cessation counselling may usefully adapt strategies that have long been trained as part of behavioral counselling for smoking cessation.

Practical counseling in this study focused on two elements:

1. Helping the participant to identify situations that have historically triggered the individual’s motivation to vape (e.g., social situations, stressful situations, negative emotions).
2. Assisting the participant to practice using a range of cognitive and behavioral coping skills in response to trigger situations.

Participants were adviced to use the following coping skills and strategies: a) adopt physical activities, possibly an exercise/sport that is fun for you like playing tennis, ultimate frisbee, taking a swing at batting cages, but even a short walk or dancing can do the trick (the endorphin boost you can get from physical activity can crush a craving); b) use a distraction to take your mind off the craving, by reading a blog/book, playing an instrument, watching TV or a movie, playing a game, doing sudokus or solving crossword puzzles, playing with a pet, texting friends (cravings will pass, if you can give them a minute or two, hence find the activities that will keep you busy for those few minutes); c) throw out anything that reminds you of vaping and that could trigger a craving by creating a temptation-free home/office (make sure your room, backpacks, purses and pockets are free of any e-cigarettes and things you need to vape); d) break behavioral habits, by replacing behavioral habits connected to vaping (e.g. starting the morning by drinking coffee and vaping an e-cigarette) with new rituals (e.g. replace morning coffee with juice or tea). Effective coping strategies may also include controlled breathing exercises (deep/slow breathing), or alternative distraction techniques (e.g. taking a shower, chewing sugar-free gum, eating fresh fruits, fiddling with a pen or squeezing a stress ball, and drinking from a water bottle).

***Promptly addressing slips and relapses***

There is very limited information about slips and early relapse when quitting vaping. However, similar to smoking cessation, vaping cessation is not a single event, but rather a process, in which relapse to vaping is a common event. Our approach was partially adapted from recommendations used for assisting smokers who relapse early in the course of their smoking cessation program (**vi**).

Slips and early relapse must be addressed promptly. Participants will have a better sense of their vaping triggers, and counselors can better focus on how to get quickly him/her back on track: “No matter what, you’ve proven that you can do this. Relapses is common, but must be used as a learning experiences through which one can better focus coping skills and improve how to get through the change process”. Slips and early relapses are best addressed by telephone counselling (**vii**). Participants are provided with contact details of their counselor for these situations. It is fundamental to identify the vaping trigger/cue so that the cessation strategy can be individualized with focused coping skills. Moreover, we reviewed whether the subject used the cessation medication in an effective manner and determine whether the drug was helpful.

**References**

1. <https://apps.who.int/iris/bitstream/handle/10665/112835/9789241506953_eng.pdf>
2. Prochaska, J. O., & DiClemente, C. C. (1983). Stages and processes of self-change of smoking: Toward an integrative model of change. Journal of Consulting and Clinical Psychology, 51(3), 390-395. http://dx.doi.org/10.1037/0022-006X.51.3.390
3. https://www.cdc.gov/tobacco/campaign/tips/quit-smoking/7-common-withdrawal-symptoms/index.html
4. West R, Ussher M, Evans M, Rashid M. Assessing DSM-IV nicotine withdrawal symptoms: a comparison and evaluation of five different scales. Psychopharmacology (Berl). 2006 Mar;184(3-4):619-27. doi: 10.1007/s00213-005-0216-z. Epub 2005 Nov 25. PMID: 16308727.
5. Liu G, Wasserman E, Kong L, Foulds J. A comparison of nicotine dependence among exclusive E-cigarette and cigarette users in the PATH study. Prev Med. 2017 Nov;104:86-91. doi: 10.1016/j.ypmed.2017.04.001. Epub 2017 Apr 4. PMID: 28389330; PMCID: PMC5868349.
6. Caponnetto P, Keller E, Bruno CM, Polosa R. Handling relapse in smoking cessation: strategies and recommendations. Intern Emerg Med. 2013 Feb;8(1):7-12. doi: 10.1007/s11739-012-0864-z. Epub 2012 Oct 7. PMID: 23054409.
7. Wu, L., He, Y., Jiang, B., Zuo, F., Liu, Q., Zhang, L., & Zhou, C. (2016). Additional follow-up telephone counselling and initial smoking relapse: a longitudinal, controlled study. BMJ open, 6(4), e010795. <https://doi.org/10.1136/bmjopen-2015-010795>
